# Automatic Speech Recognition of Conversational Speech in Individuals With Disordered Speech

**DOI:** 10.1044/2024_JSLHR-24-00045

**Published:** 2024-07-04

**Authors:** Jimmy Tobin, Phillip Nelson, Bob MacDonald, Rus Heywood, Richard Cave, Katie Seaver, Antoine Desjardins, Pan-Pan Jiang, Jordan R. Green

**Affiliations:** a Google LLC, Mountain View, CA; b MND Association, Northampton, United Kingdom; c MGH Institute of Health Professions, Boston, MA; d Harvard University, Cambridge, MA

## Abstract

**Purpose:**

This study examines the effectiveness of automatic speech recognition (ASR) for individuals with speech disorders, addressing the gap in performance between read and conversational ASR. We analyze the factors influencing this disparity and the effect of speech mode–specific training on ASR accuracy.

**Method:**

Recordings of read and conversational speech from 27 individuals with various speech disorders were analyzed using both (a) one speaker-independent ASR system trained and optimized for typical speech and (b) multiple ASR models that were personalized to the speech of the participants with disordered speech. Word error rates were calculated for each speech model, read versus conversational, and subject. Linear mixed-effects models were used to assess the impact of speech mode and disorder severity on ASR accuracy. We investigated nine variables, classified as technical, linguistic, or speech impairment factors, for their potential influence on the performance gap.

**Results:**

We found a significant performance gap between read and conversational speech in both personalized and unadapted ASR models. Speech impairment severity notably impacted recognition accuracy in unadapted models for both speech modes and in personalized models for read speech. Linguistic attributes of utterances were the most influential on accuracy, though atypical speech characteristics also played a role. Including conversational speech samples in model training notably improved recognition accuracy.

**Conclusions:**

We observed a significant performance gap in ASR accuracy between read and conversational speech for individuals with speech disorders. This gap was largely due to the linguistic complexity and unique characteristics of speech disorders in conversational speech. Training personalized ASR models using conversational speech significantly improved recognition accuracy, demonstrating the importance of domain-specific training and highlighting the need for further research into ASR systems capable of handling disordered conversational speech effectively.

Artificial Intelligence (AI) is poised to transform communication for individuals with speech disorders, marking a turning point in assistive communication technologies. Automatic speech recognition (ASR) is a powerful application of assistive AI that has the potential to mitigate debilitating communication barriers and improve access to technology for individuals with speech impairments. Speech impairments can manifest in various forms, such as slurred speech, inconsistent speech rhythm, or atypical pronunciations, which are typically not well-represented in the data sets used to train conventional ASR systems (MacDonald et al., 2021). This lack of representation leads to higher transcription error rates and reduced effectiveness in real-world applications, making these technologies less accessible to individuals with speech disorders.

Project Euphonia, a Google Research initiative, is focused on helping people with disordered speech be better understood. Since its inception in June 2018, the project has been actively amassing a large data set of disordered speech samples for the purpose of better improving disordered speech recognition (MacDonald et al., 2021). A primary objective of the research team is to improve the functionality of ASR for people with atypical speech patterns. The team recently tested the accuracy of a state-of-the-art personalized, or speaker-dependent, ASR model for recognizing a wide variety of speech impairments and severities (Green et al., 2021; Tomanek et al., 2023). This study revealed that the median word error rate (WER) for short, read phrases was approximately 5%, demonstrating the system's effective performance in accurately transcribing scripted disordered speech.

Although this level of accuracy is encouraging, these systems have not been vetted for their recognition accuracy when deployed in functional communication contexts such as conversational speech, which is widely recognized as a difficult domain. Conversational speech presents unique ASR challenges due to its distinct characteristics, such as less precise and faster articulation (DiCanio et al., 2015; Jacewicz et al., 2010; Kuo & Weismer, 2016), and the presence of irregularities such as interruptions, hesitations, repairs, and nonlexical sounds (Ward, 2006). Depending on the context, conversational speech may also include local and regional language variations and rare words (Quaglio & Biber, 2006), especially those specific to individual users such as proper nouns. In addition, in real-world applications where recording conditions are typically less controlled, issues such as fluctuating mouth-to-microphone distances, simultaneous dialogue from conversation participants, and elevated background noise could present additional challenges.

As a consequence, conversational WERs are nearly double those of scripted or read speech (see Gabler et al., 2023). This performance gap is particularly problematic because people do not read prompts when using ASR in real-life applications such as during conversations, issuing machine commands, or voice typing. Improving conversational ASR of disordered speech, therefore, is essential for addressing accessibility and communication challenges for those with speech impairments. In addition, more robust conversational ASR could significantly accelerate improvements in disordered speech ASR. For example, a model that could recognize untranscribed speech recordings of individuals with speech disorders with a reasonable degree of accuracy would allow researchers to utilize a vast amount of pseudolabeled speech recordings for semisupervised speaker-independent model training (Liao et al., 2013; Radford et al., 2023).

A major hurdle in achieving robust conversational ASR is the lack of adequate training data. Most speech disorder corpora focus on speech that is produced during reading, with scant examples of spontaneous conversation (H. Kim et al., 2008; Rudzicz et al., 2012). Obtaining annotated conversational samples is both time-intensive and expensive, with additional complexities arising from the challenges of accurately transcribing speech from individuals with speech disorders. Presumably, such errors will degrade ASR performance when they are propagated into training data sets.

In this study, we report on the performance gap between read and conversational ASR for individuals with speech disorders, particularly focusing on personalized models trained predominantly on read speech. One primary goal is to determine whether personalized, speaker-dependent models exhibit a smaller performance gap compared to publicly available unadapted, speaker-independent models that are trained primarily with recordings of typical speech. By making ASR more robust, personalized models may reduce errors from acoustic noise or training-set transcription inaccuracies, narrowing the performance difference between read and conversational speech. In open-ended conversations, where speech is more spontaneous and lacks a clear referent, the likelihood of transcription errors is higher compared to the more structured context of reading, especially for disordered speech samples. Additionally, we investigated various factors contributing to this disparity, such as the complexity of conversational speech, acoustic variables, and unique characteristics of different speech disorders. Our goal is to identify key factors influencing the performance gap, setting the foundation for more effective solutions. Lastly, we will explore the efficacy of domain-specific training (i.e., training an ASR model for a specific use-case) in improving ASR accuracy for conversational speech among individuals with speech disorders. To accomplish these objectives, we conducted an investigation utilizing recordings of both read and conversational speech from 27 individuals exhibiting disordered speech patterns.

## Method

### Participants and Speech Recordings

Recordings were obtained from 27 individuals (nine women, 18 men) with speech impairment participating in Project Euphonia's data collection program, detailed below. The 27 participants in this study were based in the United States and in the United Kingdom. Many speakers were not monolingual including L2 speakers who spoke Turkish, Russian, or German. Race/ethnicity data was not collected. Table 1 shows that 13 different etiologies were represented in the data set and that the speech impairment severities spanned from mild to severe.

**Table 1.** Prevalence of etiologies across severity of speech impairment.

Etiology	Severity			
	Mild (*n* = 5)	Moderate (*n* = 11)	Severe (*n* = 11)	Total
Amyotrophic lateral sclerosis	2	5	4	11
Anoxic brain injury		1		1
Ataxia		1		1
Cerebral palsy	1	2	2	5
Childhood-onset apraxia of speech		1		1
Cleft lip and palate			1	1
Down syndrome	1			1
Hearing impairment	1			1
Multiple sclerosis		1		1
Muscular dystrophy			1	1
Stroke		1		1
Vocal fold paralysis			2	2

*Recruitment.* Project Euphonia is a group within Google Research focusing on improving ASR for people with disordered speech. Project Euphonia partnered with organizations such as ADAPT Community Network, ALS Association, ALS Therapy Development Institute, LSVT Global, Motor Neurone Disease Association, and Team Gleason to recruit participants. All participants were 18 years or older. Data were collected only after explicit consent had been granted by individuals to provide speech samples for the purpose of research and improving speech recognition products and services. All participants were compensated for their contribution of speech recordings.

Because data were collected over multiple days and weeks, individuals were excluded from the study if their speech declined over time. Excluding speakers whose speech changed over time was important to ensure that the speech impairment severity ratings, which were assigned based on each participant's initial recording session, remained an accurate representation of their speech impairment. Declining speech was identified as speech that (a) declined by at least 10 WER percentage points over time (as estimated by an unadapted, speaker-independent model) and (b) increased in speech-language pathologist (SLP)-rated severity by at least 2 points on a 5-point Likert scale, as described in Tomanek et al. (2023). We have reported on the effects of speech decline on recognition accuracy in a prior paper (Tomanek et al., 2023).

*Data collection.* The speech recordings were acquired remotely in real-world environments using each participant's native hardware, which could be a cell phone, tablet, or computer, with or without an external microphone. Data were recorded at various sampling rates, at least 16 kHz, and no audio postprocessing, such as normalization, background noise filtering, or voice activity detection, was performed on the data. SLPs listened to a subset of recordings to determine speaker etiology and severity as well as identify participants with audio quality or recording issues. Additional details of data collection are described in a companion paper by MacDonald et al. (2021).

*Speech recordings.* For each subject, speech was recorded during two modes of speech: reading and conversing. For the reading condition, participants read phrases randomly from a screen prompt. The phrases came from different target domains, including home automation and phrases used when communicating with a caregiver. For the conversational condition, the speakers were instructed not to talk about any personally identifiable information (PII) and then recorded, while speaking extemporaneously to a familiar conversational partner or while responding to open-ended questions asked by an unfamiliar interviewer (Tobin & Tomanek, 2022). Utterance boundaries were participant-defined by when they began and ended recording. Recordings were reviewed thoroughly to ensure no PII was recorded and transcribed by SLPs. For each speaker, the read training sets included on average 3,245 utterances (*SD* = 1,925) and the conversational training sets included on average 180 utterances (*SD* = 274). For each experiment, subsets of subjects were selected based on the availability of the necessary data, as detailed below.

*Ethics statement.* This experiment was exempt from institutional review board approval process under 45 CFR 46.104(d)(3) (Exempt Research, 2018) since all participants were adults, all participants consented to have their speech recorded, and all speech recordings were scrubbed of any PII.

### Metadata

To identify factors that may contribute to the performance gap between read and conversational speech, we tested for the potential effects of 11 variables on ASR performance that were categorized as either a technical factor, linguistic factor, or impaired speech factor. These metadata were generated algorithmically or by experienced SLP.

*Technical factors metadata.* For recording in the test sets, we estimated the overall recording quality using the signal-to-noise ratio (SNR; C. Kim & Stern, 2008). The root-mean-square (RMS) of each signal was also computed to test if signal intensity had an effect on recognition accuracy.

*Linguistic factors metadata.* For each test set utterance, we estimated the number of words per utterance (word count), utterance duration in seconds (duration), and type–token ratio (TTR).

*Disordered speech metadata.* A trained SLP evaluated a subset of read and conversational utterances from each speaker. The SLP then rated them on a 5-point Likert scale (normal, mild, moderate, severe, profound) across eight speech dimensions: overall speech severity, intelligibility, articulatory function, phonatory function, resonatory function, respiratory function, speaking rate, and speech pattern consistency. These labels were selected based on previous research findings that demonstrated their influence on the accuracy of personalized ASR (Green et al., 2021).

The assessment of interrater reliability of the labels involved two SLPs independently evaluating the same subset of 64 recordings (see Table 2). To quantify their agreement, we employed two methods: firstly, we calculated the proportion of recordings where the raters' scores diverged by more than 1 point; secondly, we computed the intraclass correlations for the ratings from both SLPs. The results indicated that while most of the labels exhibited good to moderate agreement, the “Consistency” label demonstrated poor reliability (Koo & Li, 2016). Consequently, this label was excluded from subsequent analysis.

**Table 2.** Interrater agreement between the two speech-language pathologist raters for the speech labels that were used to grade the level of speech and voice impairment.

Speech label	1-pt agreement (%)	ICC (95% CI)	Interpretation
Severity	98.4	0.859 [0.769, 0.914]	Good reliability
Intelligibility	93.8	0.814 [0.693, 0.887]	Good reliability
Articulatory	92.2	0.795 [0.654, 0.877]	Good reliability
Phonatory	93.8	0.783 [0.643, 0.868]	Good reliability
Resonatory	92.2	0.536 [0.238, 0.718]	Moderate reliability
Respiratory	89.1	0.631 [0.396, 0.775]	Moderate reliability
Speaking rate	90.6	0.565 [0.281, 0.736]	Moderate reliability
Consistency	57.8	0.195 [−0.323, 0.511]	Poor reliability

*Note.* Each label was graded using a 5-point Likert scale (i.e., *normal*, *mild*, *moderate*, *severe*, and *profound*). ICC = intraclass correlation coefficient; CI = confidence interval.

### ASR

We evaluated the recognition accuracy of (a) one speaker-independent ASR system trained and optimized for typical speech and (b) 27 ASR models were personalized, one per speaker, using the speech of each of the respective 27 participants. The evaluation of these models involved two test sets collected from the same 27 speakers: one composed only of read samples and one only of conversational samples. All test sets were generated to ensure that there was no phrase overlap with the training data.

#### Unadapted ASR Model

We employed an unadapted, speaker-independent model that utilized an end-to-end ASR system. This system was based on the recurrent neural network transducer architecture, a widely studied framework (Graves, 2012). Our model's architecture included an encoder network with eight layers and a predictor network comprising two layers of unidirectional long short-term memory cells. The model processed inputs consisting of 80-dimensional log-mel filterbank energies and produced outputs as probability distributions for a vocabulary of 4,000-word pieces. For training, we used about 162,000 hr of conventional speech from Google's internal production data set (Narayanan et al., 2018). This standard model served as a baseline for comparing the performance of our personalized model, maintaining the same model architecture for consistent control. During inference, all data were resampled to 16 kHz. Additionally, automatic gain control was not utilized during inference for these experiments.

#### Personalized ASR Models

For each participant in our study, we created a personalized ASR model. The fine-tuning process was specifically designed for our adaptation needs and follows the same model training paradigm as Green et al. (2021), considering that available data per speaker was approximately 10 hr on average. To prevent overfitting and mitigate catastrophic forgetting, we limited updates to just the first five layers of the encoder rather than the entire model. To enhance the model's robustness, especially for dysarthric speech, we implemented SpecAugment (Park et al., 2019) as a regularization strategy. This involved optimizing several parameters, notably reducing frequency masking and increasing time masking, diverging from the standard settings used in typical speech processing. This adjustment was particularly effective, likely due to the unique characteristics of disordered speech, such as slower pace and reduced spectral diversity. For training, we employed the Adam optimizer (Kingma & Ba, 2015) with a low learning rate of 1e-5. Models were trained for 50,000 steps. The best checkpoints were picked using a development, or validation, set of prompted speech, of which much more was available than conversational.

### Pilot Study Examining the Effects of Model Training Using Conversational Speech Data

A preliminary study was undertaken using data from eight out of the 27 participants to evaluate the potential improvement in conversational recognition accuracy afforded by focusing training solely on conversational speech. The eight participants were included because they were the only participants that had at least 170 read and 170 conversational samples for training and testing, which is approximately the minimum of recordings deemed suitable based on our prior work (Tobin & Tomanek, 2022). These data were used to compare the outcomes of three personalized models, each created using the aforementioned architecture but with varying training data subsets: (a) a model trained exclusively on read speech (“All Read”), (b) a model trained solely on conversational speech (“All Conversational”), and (c) a model trained on an even mix of both (designated as “Split”). Like the experiments described above, the test sets were designed to ensure that there was no phrase overlap with the training data.

### WER Calculation

The accuracy of all models was estimated by calculating the WER across all of the utterances produced by each speaker for each condition (vs., e.g., the median of multiple WERs calculated at the utterance level). WER is defined as the number of deletions, substitutions, and insertion errors divided by the total number of tokens. This calculation yielded one WER estimate per subject for each model.

### Statistical Testing

We employed linear mixed-effects (LME) models to test for the fixed effects of ASR model (personalized vs. unadapted), speech mode (read or conversational), and severity (mild, moderate, severe), while also incorporating random intercepts for individual participants. Random slope models were also attempted but did not converge. Multiple comparisons were performed on LME output using the statsmodel package in Python 3. Standard boxplots, displaying the interquartile range (IQR) and median, were generated to summarize several of the findings. Whiskers extend to the minimum and maximum values, excluding outliers, which are shown as separate points.

## Results

**Figure 1.**
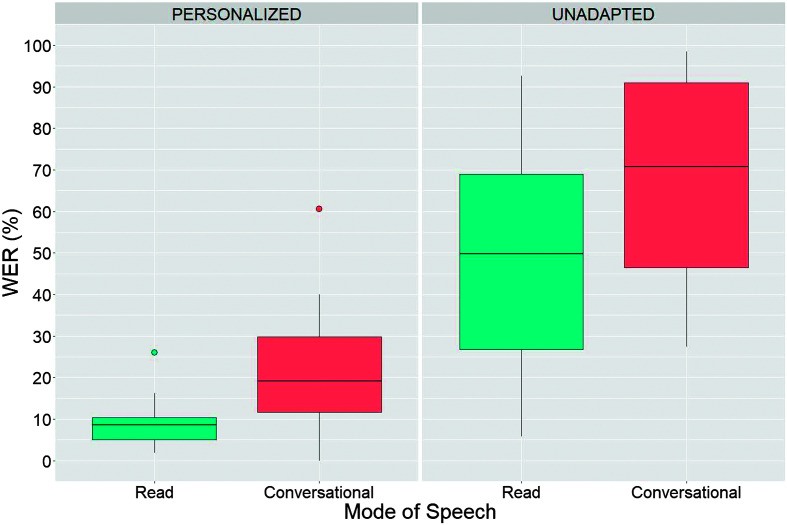
Comparative analysis of recognition accuracy of read versus conversational speech across unadapted and personalized automatic speech recognition models. WER = word error rate.

WER findings for the read and conversational speech samples across the personalized and unadapted models are presented in Figure 1. The results of the LME are presented in Table 3. The WER was significantly higher for the unadapted models (*Mdn* = 60.8, IQR [37–77]) compared to the personalized models (*Mdn* = 10.3, IQR [7–19]), *p* < .001. The WER for conversational speech (*Mdn* = 36.1, IQR [19–70]) was significantly higher than for read speech (*Mdn* = 14.6, IQR [8–48]), *p* < .001. This trend of higher WER for conversational speech was statistically significant for both personalized (*p* < .001) and unadapted models (*p* < .001).

**Table 3.** LME summary for whole sample covering WER ~ Model + Domain + Severity.

Variable	Coef.	*SE*	*z*	*P* > *|z|*	[0.025	0.975]
Intercept	0.103	6.056	0.017	.986	−11.767	11.972
Model	43.108	2.767	15.578	.001	37.684	48.532
Domain	−16.174	2.789	−5.800	.001	−21.640	−10.708
Severity	10.684	2.456	4.351	.001	5.871	15.497
Group var	45.629	2.169				

*Note.* For these analyses, severity labels were coded as a continuous variable (e.g., *mild* = 0, *moderate* = 1, and *severe* = 2). LME = linear mixed-effects; WER = word error rate; Coef. = coefficient; *SE* = standard error.

**Figure 2.**
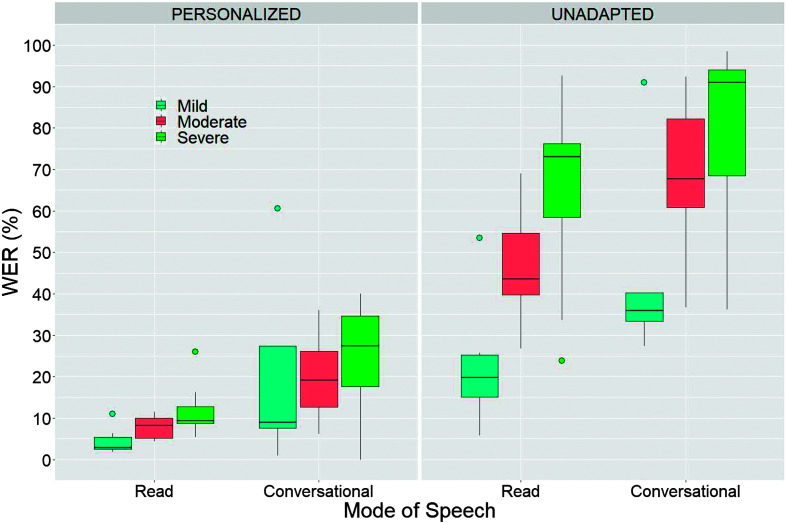
Recognition accuracy of read and conversational speech as a function of speech impairment severity for the personalized and unadapted models. WER = word error rate.

As shown in Figure 2, for the unadapted models, speech impairment severity significantly degraded recognition accuracy for both read (*p* < .001) and conversational utterances (*p* = .003). In contrast, within the personalized models, the severity of speech impairment significantly degraded the recognition accuracy for read utterances (*p* < .001) but not for conversational utterances (*p* = .643). Thus, as depicted in Figure 2, although there was a trend for personalized WERs to increase for both the read and conversational conditions, a statistical effect of severity was not detected for the conversational condition due to the large variance across subjects and the small median differences (e.g., 3.55 WER).

### Factors Accounting for Performance Gap Between Read and Conversational Speech

**Figure 3.**
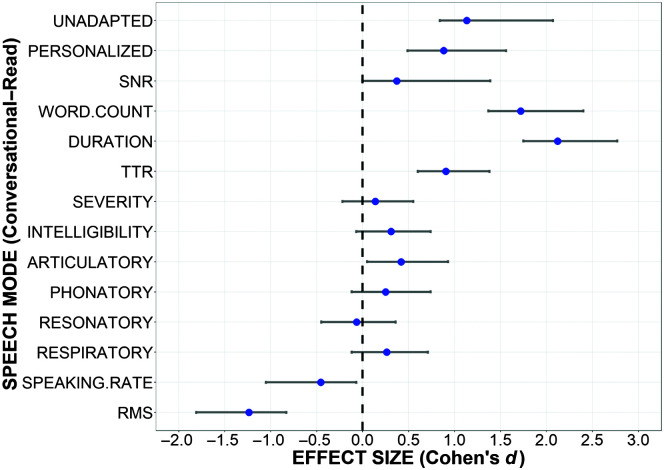
Effect sizes analysis of variables thought to impact automatic speech recognition accuracy. SNR = signal-to-noise ratio; TTR = type–token ratio; RMS = root-mean-square of signal amplitude.

An effect size analysis was conducted to estimate the impact of each variable on the performance gap between conversational and read speech (see Figure 3). For each variable, the effect sizes were based on differences between the speaking conditions (conversational–read) such that a positive effect indicates higher values for conversational speech. The linguistic variables had large effects on the WERs; the utterances were longer and more varied (TTR) in conversation than in reading. The SNR was similar in both speaking modes. Some of the speech disorder related factors had moderate effects. Speech severity was judged as similar across speech modes, but speech was less accurate and more inconsistent in conversation. The speaking rate was judged as less impaired in conversation than in reading, which could be a secondary effect of an increased rate during conversation.

### Pilot Study Examining the Effects of Model Training Using Conversational Speech (Data From Eight Participants)

**Figure 4.**
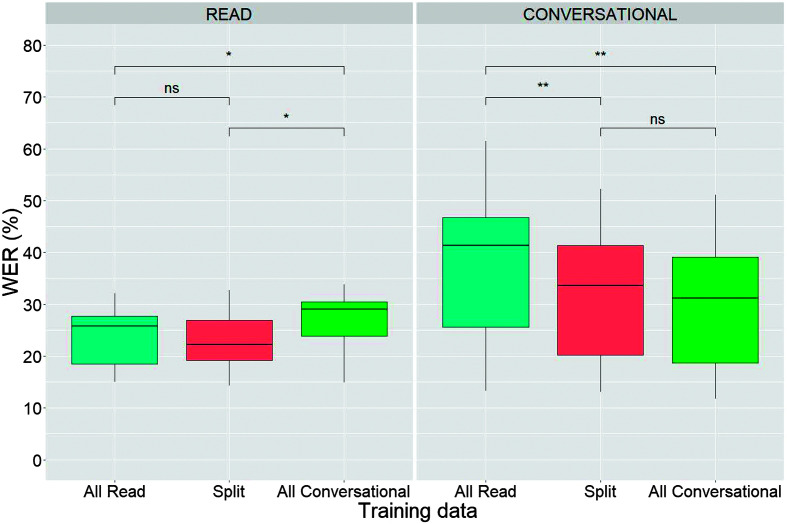
Result of pilot study conducted to determine the differential impact of training on all read or all conversational utterances when testing on the read and conversational speech test sets. The split training data contained 50% read and 50% conversational samples. Statistical significance codes: *p = .05; **p = .01; ns = not significant. WER = word error rate.

Figure 4 presents a boxplot comparison of WERs across the three different training data conditions, for the read (left panel) and conversational utterances (right panel). For read utterances, there was no significant difference in WER between systems trained on all read data and those trained on a split of read and conversational data, while a significant increase in WER was observed for systems trained on all conversational data (*p* < .01). In contrast, for the conversational utterances, systems trained on all read data exhibit a significantly higher (*p* < .001) WER compared to those trained on a mix or all conversational data, with no statistically significant difference noted between the mixed training and all conversational training conditions.

## Discussion

Although speech recognition technology offers significant potential to improve the lives of many individuals with speech disabilities, its current accuracy rates frequently fall short, making these systems particularly ineffective for real-world applications among this population. Since 2018, the Euphonia team has been pursuing a research program to make ASR technology more effective and inclusive, so everyone has the opportunity to communicate in person and with the digital world. The current investigation sought to extend this research by evaluating the accuracy of unadapted and personalized models when used by individuals with speech impairments for functional communication, specifically, in conversation. The recognition of conversational speech is a demanding task for current ASR, yet critical for improving accessibility and communication for those with speech impairments. Our evaluation of accuracy was relative to the performance of these models on recognizing read speech, which is the type of speech most often available for training disordered speech recognition models.

Overall, our findings revealed substantial degraded recognition of conversational speech relative to read speech by 10 to 20 points for the personalized and unadapted speech recognition models, respectively. However, personalization led to significant gains in conversational speech recognition accuracy bringing median WERs down by as much as 62 percentage points (e.g., from 90% to 28% for the group with severe speech impairments). When utilizing personalized models, the WERs showed a decrease as the severity of impairment increased for the read condition. However, this pattern was less pronounced and not statistically significant for the conversational condition. The broad dispersion of boxplots for conversational speech, as seen in the left panel of Figure 2, indicates that the WERs for personalized conversational models exhibited greater variability than those recorded in the read condition. This heightened variability in conversational speech WER is likely attributable to the inherent fluctuations found in conversational speech, including differences in accuracy, rate, loudness, and linguistic complexity. These results align with previous studies showing that ASR accuracy is lower for disordered speech than for typical speech and degrades as a function of severity (Gutz et al., 2022; Tomanek et al., 2023).

The effect size study was an exploration of the potential factors that degrade conversational ASR performance. The linguistic variables (i.e., TTR, word count, and duration) appeared to have the greatest differences across speaking modes with our conversational samples characterized by increased lexical diversity and longer utterances. In addition, our findings indicated that some atypical speech characteristics were more pronounced in conversational than in read speech. Specifically, while overall severity and intelligibility remained relatively constant across the two modes of speech, articulatory impairments were judged to be more severe in conversation, and speech intensities (RMS) were reduced. The finding that articulatory impairments are more pronounced during conversational speech, as opposed to read speech, is consistent with prior research showing that in healthy controls and individuals with speech disorders, speech motor performance declines as cognitive, linguistic, or motor demands of a task escalate (Dragicevic et al., 2023; Fournet et al., 2022; Kuruvilla-Dugdale et al., 2018). These findings are also consistent with the findings by Allison et al. (2019) that speech intelligibility worsens with increased utterance length in persons with dysarthria due to ALS. Surprisingly, speaking rate was judged to be less affected in conversation, possibly due to faster speech production in spontaneous speech (Jacewicz et al., 2010). Despite expectations of more background noise in conversation, the audio quality of read speech samples was lower than for conversational speech samples, as indexed by SNR. When taken together, these insights highlight the challenges in conversational ASR for disordered speech, suggesting the need for further controlled studies to explore these factors more comprehensively.

The findings from the conversational model training experiment demonstrated that despite all of the irregularities of conversational speech, significant gains in recognition accuracy can be achieved when conversational speech samples are included in the training data. These findings underscore the necessity of incorporating conversationally produced, linguistically diverse stimuli in model training to address accuracy issues in disordered speech more effectively for real-world applications. An important finding from the pilot experiment was that incorporating read speech into the training process did not negatively affect the recognition of conversational speech. This finding suggests that a significant portion of the training set, even up to 50% in this case, can include other speech modes without compromising the effectiveness of conversational speech recognition.

### Limitations

A key limitation of this research is the restricted diversity in the speech impairment types within the study sample, with the majority consisted of individuals with dysarthria from ALS or CP (see Table 1). This lack of variety limits the generalizability of the findings to other speech disorders. Future research could benefit from including a wider array of speech impairments and a larger participant pool to enhance the robustness and applicability of the findings.

### Future directions

Project Euphonia's goal is to improve speech recognition for people with disordered speech. The team continues to collect data with a focus on non-English data. Future experiments can confirm current personalization recommendations for ASR models in other languages such as Japanese, French, Spanish, or Hindi. Although the current study was focused on evaluating model performance based on WER, functional ASR communication systems should also be evaluated on other factors such as the influence of transcription errors on the conveyed meaning, the overall user experience, and user adoption rates. Prioritizing the minimization of semantic errors is as important as reducing WER to preserve the meaning of what a speaker is trying to communicate (Tomanek et al., 2024). Moreover, the improvement of recognition capabilities for conversational speech, particularly through the integration of large language models, is essential. Also, the incorporation of multimodal signals, such as contextual cues and facial or lip movements, offers a promising avenue to further bolster recognition accuracy. Finally, future directions for speech recognition research should also focus on enhancing out-of-the-box performance for disordered speech without relying on extensive personalization. Speaker-independent models, such as Google's Universal Speech Model, have been shown to improve with the inclusion of Euphonia data (Tobin & Tomanek, 2023). Further experiments in non-English languages would improve accessibility globally. Moving forward, it is critical to explore these caveats and chart a path for future research that will make speech recognition tools more inclusive and effective for users with speech impairments.

## Data Availability Statement

The data sets presented in this article are not available to be shared publicly in adherence to the written consent of Project Euphonia participants. Individual contributors to Project Euphonia may request their data by e-mailing euphonia-project@google.com.

## Notes

**Publisher Note:** This article is part of the Forum: Research Symposium on Artificial Intelligence.

## References

[bib1] Allison, K. M., Yunusova, Y., & Green, J. R. (2019). Shorter sentence length maximizes intelligibility and speech motor performance in persons with dysarthria due to amyotrophic lateral sclerosis. American Journal of Speech-Language Pathology, 28(1), 96–107. 10.1044/2018_AJSLP-18-0049 31072158 PMC6503867

[bib3] DiCanio, C., Nam, H., Amith, J. D., García, R. C., & Whalen, D. H. (2015). Vowel variability in elicited versus spontaneous speech: Evidence from Mixtec. Journal of Phonetics, 48, 45–59. 10.1016/j.wocn.2014.10.003

[bib4] Dragicevic, D. A., Dahl, K. L., Perkins, Z., Abur, D., & Stepp, C. E. (2023). Effects of a concurrent working memory task on speech acoustics in Parkinson's disease. American Journal of Speech-Language Pathology, 33(1), 418–434. 10.1044/2023_AJSLP-23-00214 38081054 PMC11001185

[bib2] Exempt Research, 45 CFR 46.104(d)(3). (2018). https://www.ecfr.gov/on/2018-07-19/title-45/section-46.104

[bib5] Fournet, M., Chiuvé, S. C., & Laganaro, M. (2022). Attentional demand of motor speech encoding: Evidence from Parkinson's disease. Journal of Speech, Language, and Hearing Research, 65(10), 3758–3775. 10.1044/2022_JSLHR-22-00096 36201164

[bib6] Gabler, P., Geiger, B. C., Schuppler, B., & Kern, R. (2023). Reconsidering read and spontaneous speech: Causal perspectives on the generation of training data for automatic speech recognition. Information, 14(2), Article 137. 10.3390/info14020137

[bib7] Graves, A. (2012). Sequence transduction with recurrent neural networks. Springer. 10.1007/978-3-642-24797-2

[bib8] Green, J. R., MacDonald, R. L., Jiang, P.-P., Cattiau, J., Heywood, R., Cave, R., Seaver, K., Ladewig, M., Tobin, J., Brenner, M. P., Nelson, P. C., & Tomanek, K. (2021). Automatic speech recognition of disordered speech: Personalized models outperforming human listeners on short phrases. Proceedings of Interspeech, 4778–4782. 10.21437/Interspeech.2021-1384

[bib9] Gutz, S. E., Stipancic, K. L., Yunusova, Y., Berry, J. D., & Green, J. R. (2022). Validity of off-the-shelf automatic speech recognition for assessing speech intelligibility and speech severity in speakers with amyotrophic lateral sclerosis. Journal of Speech, Language, and Hearing Research, 65(6), 2128–2143. 10.1044/2022_JSLHR-21-00589 PMC9567308 35623334

[bib10] Jacewicz, E., Fox, R. A., & Wei, L. (2010). Between-speaker and within-speaker variation in speech tempo of American English. The Journal of the Acoustical Society of America, 128(2), 839–850. 10.1121/1.3459842 20707453 PMC2933259

[bib12] Kim, C., & Stern, R. M. (2008). Robust signal-to-noise ratio estimation based on waveform amplitude distribution analysis. Proceedings of Interspeech, 2598–2601. 10.21437/Interspeech.2008-644

[bib11] Kim, H., Hasegawa-Johnson, M., Perlman, A., Gunderson, J., Huang, T. S., Watkin, K., & Frame, S. (2008). Dysarthric speech database for universal access research. Proceedings of Interspeech, 1741–1744. https://doi.org/10.21437/Interspeech.2008-480

[bib13] Kingma, D., & Ba, J. (2015). Adam: A method for stochastic optimization. In Proceedings of the 3rd International Convention on Learning Representations (ICLR 2015).

[bib14] Koo, T. K., & Li, M. Y. (2016). A guideline of selecting and reporting intraclass correlation coefficients for reliability research. Journal of Chiropractic Medicine, 15(2), 155–163. 10.1016/j.jcm.2016.02.012 27330520 PMC4913118

[bib15] Kuo, C., & Weismer, G. (2016). Vowel reduction across tasks for male speakers of American English. The Journal of the Acoustical Society of America, 140(1), 369–383. 10.1121/1.4955310 27475161 PMC6910000

[bib16] Kuruvilla-Dugdale, M., Custer, C., Heidrick, L., Barohn, R., & Govindarajan, R. (2018). A phonetic complexity-based approach for intelligibility and articulatory precision testing: A preliminary study on talkers with amyotrophic lateral sclerosis. Journal of Speech, Language, and Hearing Research, 61(9), 2205–2214. 10.1044/2018_JSLHR-S-17-0462 PMC6195044 30208408

[bib17] Liao, H., McDermott, E., & Senior, A. (2013). Large scale deep neural network acoustic modeling with semi-supervised training data for YouTube video transcription. 2013 IEEE Workshop on Automatic Speech Recognition and Understanding, 368–373.

[bib18] MacDonald, B., Jiang, P. P., Cattiau, J., Heywood, R., Cave, R., Seaver, K., Ladewig, M. A., Tobin, J., Brenner, M. P., Nelson, P. C., Green, J. R., & Tomanek, K. (2021). Disordered speech data collection: Lessons learned at 1 million utterances from Project Euphonia. Proceedings of Interspeech 2021, 4833–4837. 10.21437/Interspeech.2021-697

[bib19] Narayanan, A., Misra, A., Sim, K. C., Pundak, G., Tripathi, A., Elfeky, M., Haghani, P., Strohman, T., & Bacchiani, M. (2018). Toward domain-invariant speech recognition via large scale training. Proceedings of the IEEE Spoken Language Technology Workshop, 441–447.

[bib20] Park, D. S., Chan, W., Zhang, Y., Chiu, C. C., Zoph, B., Cubuk, E. D., & Le, Q. V. (2019). Specaugment: A simple data augmentation method for automatic speech recognition. Proceedings of Interspeech 2019, 2613–2617. 10.21437/Interspeech.2019-2680

[bib21] Quaglio, P., & Biber, D. (2006). The grammar of conversation. In B. Aarts & A. McMahon (Eds.), The handbook of English linguistics (pp. 692–723). Blackwell Publishing. 10.1002/9780470753002.ch29

[bib22] Radford, A., Kim, J. W., Xu, T., Brockman, G., McLeavey, C., & Sutskever, I. (2023). Robust speech recognition via large-scale weak supervision. Proceedings of the 40th International Conference on Machine Learning, 28492–28518. https://proceedings.mlr.press/v202/radford23a/radford23a.pdf [PDF]

[bib23] Rudzicz, F., Namasivayam, A. K., & Wolff, T. (2012). The TORGO database of acoustic and articulatory speech from speakers with dysarthria. Language Resources and Evaluation, 46(4), 523–541. 10.1007/s10579-011-9145-0

[bib24] Tobin, J., & Tomanek, K. (2022). Personalized automatic speech recognition trained on small disordered speech datasets. ICASSP 2022–2022 IEEE International Conference on Acoustics, Speech and Signal Processing (ICASSP), 6637–6641. 10.1109/ICASSP43922.2022.9747516

[bib25] Tobin, J., & Tomanek, K. (2023, June 21). Responsible AI at Google Research: AI for social good. Google Research Blog. https://research.google/blog/responsible-ai-at-google-research-ai-for-social-good/

[bib26] Tomanek, K., Seaver, K., Jiang, P. P., Cave, R., Harrell, L., & Green, J. R. (2023). An analysis of degenerating speech due to progressive dysarthria on ASR performance. ICASSP 2023–2023 IEEE International Conference on Acoustics, Speech and Signal Processing (ICASSP), 1–5. 10.1109/ICASSP49357.2023.10097195

[bib27] Tomanek, K., Tobin, J., Venugopalan, S., Cave, R., Seaver, K., Green, J. R., & Heywood, R. (2024). Large language models as a proxy for human evaluation in assessing the comprehensibility of disordered speech transcription. ICASSP 2024–2024 IEEE International Conference on Acoustics, Speech and Signal Processing (ICASSP), 10846–10850. 10.1109/ICASSP48485.2024.10447177

[bib28] Ward, N. (2006). Non-lexical conversational sounds in American English. Pragmatics & Cognition, 14(1), 129–182. 10.1075/pc.14.1.08war

